# Seasonal and sex-dependent gene expression in emu (*Dromaius novaehollandiae)* fat tissues

**DOI:** 10.1038/s41598-022-13681-5

**Published:** 2022-06-08

**Authors:** Kristina Wright, Ka Ming Nip, Ji Eun Kim, Kimberly M. Cheng, Inanc Birol

**Affiliations:** 1 Canada’s Michael Smith Genome Sciences Centre, BC Cancer, 570 West 7th, Avenue, Vancouver, BC V5Z 4S6 Canada; 2 Avian Research Centre, Faculty of Land and Food Systems, University of British Columbia, 2357 Main Mall, Vancouver, BC V6T 1Z4 Canada

**Keywords:** Gene regulatory networks, Computational biology and bioinformatics, Gene ontology

## Abstract

Emu (*Dromaius novaehollandiae*) farming has been gaining wide interest for fat production. Oil rendered from this large flightless bird’s fat is valued for its anti-inflammatory and antioxidant properties for uses in therapeutics and cosmetics. We analyzed the seasonal and sex-dependent differentially expressed (DE) genes involved in fat metabolism in emus. Samples were taken from back and abdominal fat tissues of a single set of four male and four female emus in April, June, and November for RNA-sequencing. We found 100 DE genes (47 seasonally in males; 34 seasonally in females; 19 between sexes). Seasonally DE genes with significant difference between the sexes in gene ontology terms suggested integrin beta chain-2 (*ITGB2*) influences fat changes, in concordance with earlier studies. Six seasonally DE genes functioned in more than two enriched pathways (two female: angiopoietin-like 4 (*ANGPTL4*) and lipoprotein lipase (*LPL*); four male: lumican (*LUM*), osteoglycin (*OGN*), aldolase B (*ALDOB*), and solute carrier family 37 member 2 (*SLC37A2*)). Two sexually DE genes, follicle stimulating hormone receptor (*FSHR*) and perilipin 2 (*PLIN2*), had functional investigations supporting their influence on fat gain and loss. The results suggested these nine genes influence fat metabolism and deposition in emus.

## Introduction

*Dromaius novaehollandiae,* commonly known as emu, is a large, flightless bird native to Australia, and is farmed worldwide^1^. Emus are farmed for meat, skin, and oil that is rendered from their abdominal and back fat^2^. Emu oil, traditionally used by Australian aboriginals for wound healing, is generally not added to food, but it is sold commercially for uses in therapeutics and cosmetics, due to its wound reparative properties^3,4^. Various studies have demonstrated the oil’s anti-inflammatory and antioxidant properties, which have been proposed to originate from compounds such as n-3 and n-9 fatty acids, carotenoids, and flavones^4,5^. Emus go through an annual cycle of fat gain and fat loss^6^. In spring and summer, they gain fat when food is plentiful to sustain themselves during winter when food is scarce^6^. The birds adapted their cycles to local seasons. In Canada, emus start gaining fat in April, continue gaining fat in June, and start losing fat in November when male and female emus breed. Females and males are usually paired in a 1:1 ratio, although females may have successive mates in a mating system known as sequential polyandry^7^. The male incubates the eggs with little or no food intake or water for eight weeks, losing up to 25% of his body weight, and continues to care for the hatched chicks^7,8^. Substantial fat gain and loss through seasons is not unique to emus; it is also seen in migratory birds. During migratory flights, birds must expend their accumulated fat stores to oxidize triacylglycerols in adipose tissue as their main fuel of energy for their long expedition^9^.

Fat tissue is a major energy reserve in many animals, including birds, and has an additional function as an endocrine organ releasing hormones called adipokines to control lipid homeostasis^10,11^. When nutrients are abundant, triacylglycerols are stored as energy in adipose tissue, and during times of starvation, its conversion into free fatty acids makes this energy available for use throughout the body^11,12^. Adipose tissue is heterogenous; it is mainly composed of mature adipocytes, and other cell types including fibroblasts, endothelial cells, pericytes, nerve cells, pre-adipocytes, and macrophages^12,13^. While it is known that emu back and abdominal fat composition shows variability by age, sex, and fat tissue location, the mechanisms behind fat accumulation is still poorly understood^2^.

Genomic analyses have been used in chicken meat quality traits selection to identify candidate genes or mutations^14^. Expression analyses are also valuable to clarify the complex biology behind meat quality traits and to potentially improve the genomic selection of chicken^14^. Here we investigate via RNA-seq and bioinformatics approaches what genes affect emu fat metabolism and deposition. The lack of studies on the functional characterization of genes affecting emu fat storage and lack of molecular markers for use in selective breeding to improve fat production motivated this research. Our study examines the seasonal and sex-dependent expression of genes that are associated with back and abdominal fat deposition in emu fat tissues. The identification of these genes would be valuable in future studies aimed at improving emu fat production.

## Methods

Methods are reported in accordance with ARRIVE guidelines (https://arriveguidelines.org). All experiments were performed in accordance with protocols reviewed and approved by the University of British Columbia Animal Care Committee (Certificate # A10-0106).

### Animal tissue

Animal tissues were collected from 8 adult emus (4 males, 4 females) sampled at three time points (April, June, and November 2012), as described earlier^15^.

### RNA extraction and cDNA library preparation

RNA from the 24 animal tissues were extracted to build cDNA sequencing libraries as described in^16^.

### Sequencing on Mi-Seq and preprocessing of raw reads

Illumina Mi-Seq system (Illumina, SanDiego, CA), was used for sequencing the emu fat transcriptome library using Sequencing by Synthesis (SBS) technology. The 24 libraries were run in one go following manufacturer’s instructions using the Mi-Seq Reagent Kit v2 (Illumina, SanDiego, CA), with 2 × 150 PE sequencing. Mi-Seq Control Software 2.2.0-RTA 1.17.28.0—CASAVA-1.8.2 was used to generate paired-end and single-end data in FastQ format^17^. Raw reads were filtered with Q20 quality trimming to remove low quality reads with average quality score < 20 and trimming of low-quality bases from the end of reads^16^. Sequence pre-processing software Trimmomatic (v0.30)^18^, was used to obtain clean paired-end and single-end Mi-Seq data in a FastQ format, which was also subjected to quality control using FastQC. The high-quality, filtered reads were used for downstream analyses.

### Transcript quantification

Salmon (v1.0.0)^19^ was used to quantify gene-level expression based on the Ensembl^20^ Emu reference annotation (release 102) with Seqbias option.

### Differential expression analysis

DESeq2 R package (v1.26.0)^21^ was used to perform differential gene expression analysis for a total of nine pairwise comparisons between the three time points (April, June, and November) for both sexes. Seasonally DE genes were selected to be both substantially down-regulated and up-regulated genes (− 1 > log_2_(fold change) or log_2_(fold change) > 1) and survive a statistical significance threshold (Benjamini–Hochberg corrected p-value < 0.01).

A second differential gene expression analysis was executed for three pairwise comparisons between sexes for each of the time points (April, June, and November). Sexually DE genes were selected to survive substantial fold change limits (− 1 > log_2_(fold change) or log_2_(fold change) > 1) and a statistical significance threshold (Benjamini–Hochberg corrected p-value < 0.01).

### Gene ontology analysis

Gene ontology analyses were performed using gprofiler2 R package (v0.2.0)^22^ with the Ensembl database (v102)^20^ for gene annotations. *Gallus gallus*, commonly known as the chicken, acted as a reference in the analyses, because gene ontology annotation is not available for the emu, and chicken was the phylogenetically closest to emus out of all available species in Ensembl. According to TimeTree, the evolutionary distance between the chicken and emu is 111.4 MYA (retrieved May 15, 2021)^23^. Retrieved gene ontology terms were collated for all annotated expressed genes and seasonally DE genes. Categories of gene ontology terms were collected under the umbrella of three domains of classification: (1) biological processes, which refer to the molecular activities that occur within cells, tissues, and organs; (2) molecular functions, which include molecular events, such as enzymatic activities; and (3) cellular components, which describe where the transcript is active in the cell or its extracellular environment.

**Figure 1:**
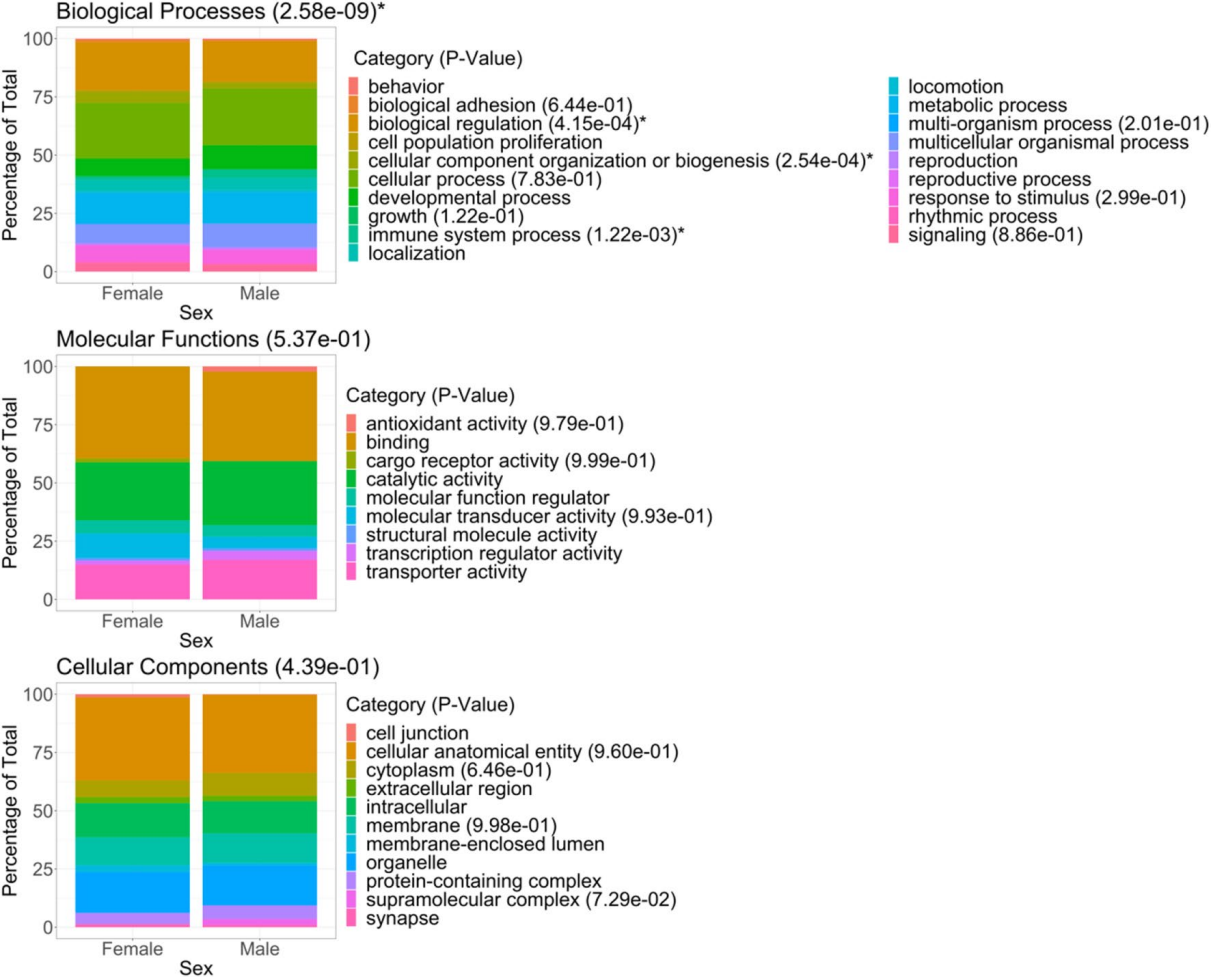
Gene ontology classification of seasonally differentially expressed genes for each sex. The two series of stacked bar plots correspond to all female and male seasonally differentially expressed transcripts. Sidak corrected p-values from χ^2^ tests for each domain and their categories within are in brackets where those significantly different between sexes are indicated with an asterisk. Those without p-values were equal to 1 and, therefore, were not significantly different between sexes.

A series of χ^2^ tests^24^ were performed to determine the statistical similarity of the relative frequencies of categories between sexes. A Sidak^25^ correction factor of 6 was used for the three domains of classification belonging to the two sets of comparisons of all and DE genes. A Sidak correction factor of 83 was used for the number categories present in both sets of comparisons of all and DE genes (Fig. 1, Supplementary Fig. S1). Sidak corrected p-values less than 0.05 were considered to be statistically significant.

### Pathway analysis

Pathway analysis was performed using gprofiler2 R package (v0.2.0)^22^, which utilizes the Reactome database^26^ (gprofiler2 archive based on Ensembl database v102). Again, the chicken reference and annotations were used for analysis. Emu transcripts were mapped to the chicken gene annotation using orthology data from the Ensembl database that was accessed via the biomaRt R package^27^. Female and male annotated seasonally DE genes were inputted individually for two sets of enrichment analyses. Significantly enriched pathways survived a Benjamini–Hochberg false discovery rate threshold of 5% and pathways containing less than 6 genes were removed.

In order to establish statistical similarity of the relative gene response (not observed, expressed but not DE, and DE) between sexes, a series of χ^2^ tests^24^ were performed. A Sidak^25^ correction factor of 19 was used for the total number of enriched pathway profile comparisons (10 female and 9 male) (Supplementary Figs. S2, S3). Sidak corrected p-values less than 0.05 were considered statistically significant.

### PathVisio

We used PathVisio (v3.3.0)^28^, which utilizes the WikiPathways database (v20210528)^29^, to generate two chicken triacylglycerol synthesis pathway visualizations for females and males. Gene products were coloured according to their Benjamini–Hochberg corrected p-values from the female seasonal differential expression analysis. The pathway visualization displays three female seasonally DE genes expression levels by their log_2_TPM values calculated by DESeq2^21^ for each month.

The R scripts for our differential expression analysis, gene ontology analysis, and pathway analysis are publicly available on GitHub at: https://github.com/bcgsc/emu_rnaseq.

### Ethics approval

All experiments were performed in accordance with protocols reviewed and approved by the University of British Columbia Animal Care Committee (Certificate # A10-0106). All experiments were performed in accordance with relevant guidelines and regulations.

## Results

### Seasonal differential expression analysis

**Figure 2:**
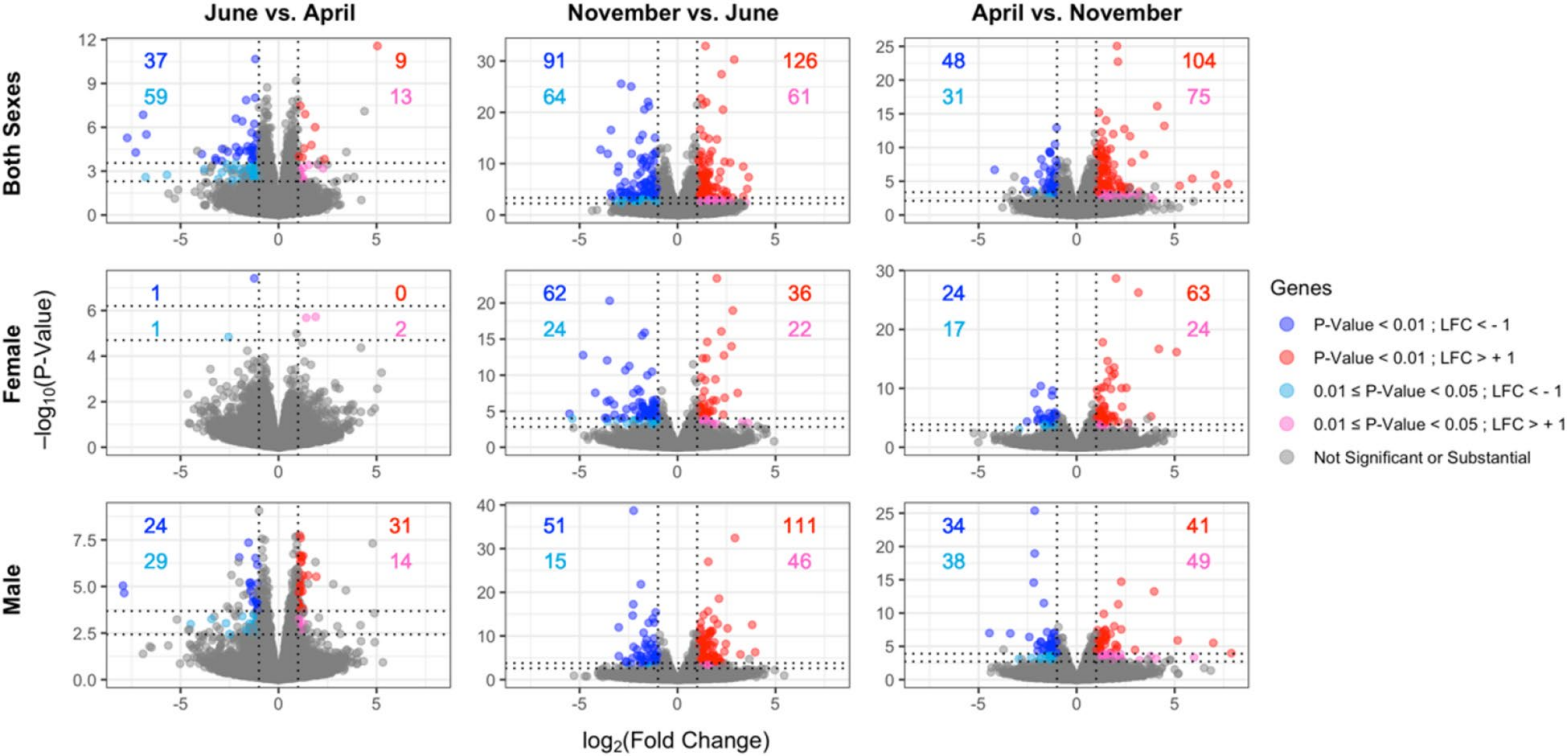
Seasonal differential expression between time points for both sexes, females, and male emus. Volcano plots of month-wise comparisons for each sex condition are displayed. Genes surviving cut offs of − 1 > log_2_(fold change) > 1 correspond to down-regulated genes (dark and light blue) and up-regulated genes (red and pink), and Benjamini–Hochberg corrected p-value < 0.01 (dark blue and red), or Benjamini–Hochberg corrected p-value between 0.01 and 0.05 (light blue and pink) are displayed. The number of genes associated with these classes is coloured for each condition of p-value and log_2_(fold change) (LFC) cutoffs within each comparison. Grey points indicate genes that either did not survive the thresholds and, therefore, were not significant, or those that were deemed non-substantial as determined by DESeq2^21^, as described in the text.

Differential expression analysis was performed to report significant changes in gene expression between time points when fat levels fluctuate. Nine pairwise comparisons were examined between the three time points (June vs. April, November vs. June, April vs. November) for both sexes, as well as for females and males separately, using DESeq2^21^. We required seasonally DE genes to survive a statistical significance threshold of Benjamini–Hochberg corrected p-value < 0.01. To account for presumed cyclic nature of seasonal variations, we also required that DE genes are both substantially down-regulated and up-regulated (− 1 > log_2_(Fold Change) > 1), using the April gene expression profiles from one year as a surrogate for the profiles of the next year. We identified 34 (47) DE genes in females (males) (Fig. 2, Supplementary File 2). Genes with non-substantial expression changes were determined by DESeq2, when expression levels had zero counts, low mean normalized counts, or were outliers as detected by Cook’s distance^21^.

### Gene ontology analysis

To better understand how the DE gene products contribute to biological systems within each sex, gene ontology analysis was performed. The R package gprofiler2^22^ analyzed under three domains of gene ontology terms (biological processes, molecular functions, and cellular components) in each sex. All female and male expressed genes were analyzed and the proportions of each category of gene ontology terms within each domain for each sex were plotted (Supplementary Fig. S1). We observed the gene ontology profiles for all expressed genes were not significantly different between the two sexes for each of the domains and all their categories within (Sidak corrected χ^2^ p-value threshold of 0.05)^24,25^.

Next, gene ontology analysis was performed on annotated seasonally DE genes (27 female, 35 male) for each sex. The proportions of gene ontology categories within each domain for females and males are shown in Fig. 1. We observed that gene ontology profiles for seasonally DE genes showed significant difference between the sexes for biological processes, but not in the domains or their respective categories for molecular functions and cellular components (Sidak corrected χ^2^ p-value threshold of 0.05). Of the 19 categories within biological processes, only three categories were significantly different between the sexes: biological regulation, cellular component organization or biogenesis, and immune system process. Notably, out of the three categories, immune system process was the only one with a higher category proportion for the male sex. Seasonally DE genes generating the gene ontology terms in immune system process for males were leucine rich repeat containing 17 (*LRRC17*) (21 terms) and integrin beta chain-2 (*ITGB2*) (41 terms), and for females was *LRRC17* (21 terms). Of the genes that comprised gene ontology terms in immune system process, *ITGB2* was the only gene present in males alone. *ITGB2* is observed to be expressed significantly higher in November when compared to June (up-regulated) and expressed significantly lower in April when compared to November (down-regulated).

### Pathway analysis

Pathway enrichment analysis can provide insight into the biological mechanisms in which the DE gene products function. Using the R package gprofiler2^22^, pathway analysis on the seasonally DE genes for each sex revealed 10 significantly enriched pathways in females and 9 in males (Supplementary Figs. S2, S3).

We observed one pathway that was shared in both female and male enriched pathways, triglyceride metabolism. We identified one enriched pathway gene response profile that was significantly different between the sexes for females: glutathione synthesis and recycling (Sidak corrected χ^2^ p-value threshold of 0.05) (Supplementary Fig. S2). For males, two enriched pathway gene response profiles were significantly different between the sexes: keratan sulfate degradation and integrin cell surface interactions (Sidak corrected χ^2^ p-value threshold of 0.05) (Supplementary Fig. S3).

### Genes seasonally differentially expressed in multiple pathways

A single gene can play a role in multiple biological processes, molecular functions, or pathways^30^. These multifunctional genes involved in multiple pathways are more likely to have essential tissue functions and are often associated with diseases^30^. Therefore, it is logical to assume that seasonally DE emu genes that participate in multiple pathways are more likely to have essential adipose tissue functions related to fat gain and loss. We examined seasonally DE genes that participated in multiple enriched pathways within each sex. We identified two genes in females functioning in multiple female-enriched pathways: angiopoietin-like 4 (*ANGPTL4*) and lipoprotein lipase (*LPL*). *ANGPTL4* was expressed significantly higher in November when compared to June (up-regulated) and expressed significantly lower in April when compared to November (down-regulated), while *LPL* expression levels showed the inversed pattern. For males, four seasonally DE genes function in multiple enriched pathways: lumican (*LUM*), osteoglycin (*OGN*), aldolase B (*ALDOB*), and solute carrier family 37 member 2 (*SLC37A2*). *LUM*, *OGN*, and *ALDOB* were expressed significantly lower in November when compared to June (down-regulated) and expressed significantly higher in April when compared to November (up-regulated), while *SLC37A2* expression levels showed the inversed pattern. The distribution of the number of times a gene appears in a pathway is in Supplementary Fig. S4.

### Sexual differential expression analysis

**Figure 3:**
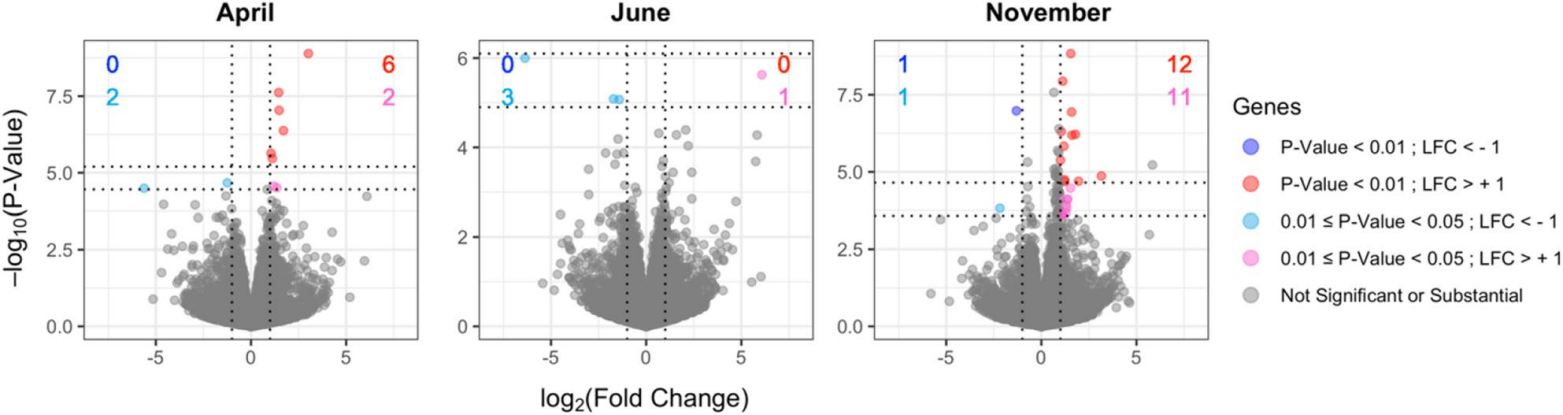
Sexually differential expression pairwise comparisons between sexes for each time point. Volcano plots of sex-wise comparisons for each month are displayed. LFC is the log_2_(fold change) of gene expression. Other plot details are the same as in Fig. 2.

To better investigate the differences in gene expression patterns between sexes, an additional differential expression analysis was performed using DESeq2^21^. Three pairwise comparisons were examined between sexes (male vs. female) for each month (April, June, and November), revealing 19 sexually DE genes (Fig. 3, Supplementary File 2). Significantly sexually DE genes were genes surviving a Benjamini–Hochberg corrected p-value threshold of 0.01. November had the highest amount (13) of sexually DE genes compared to April (6) and June (0) (Fig. 3). Based on reported functions in various studies of the annotated sexually DE genes (17), follicle stimulating hormone receptor (*FSHR*) and perilipin 2 (*PLIN2*) appear to be important genes in fat storage and breakdown. *FSHR* was expressed significantly higher in males than females in April, while *PLIN2* was expressed significantly higher in males than females in November.

### Triacylglycerol synthesis pathway visualization

**Figure 4:**
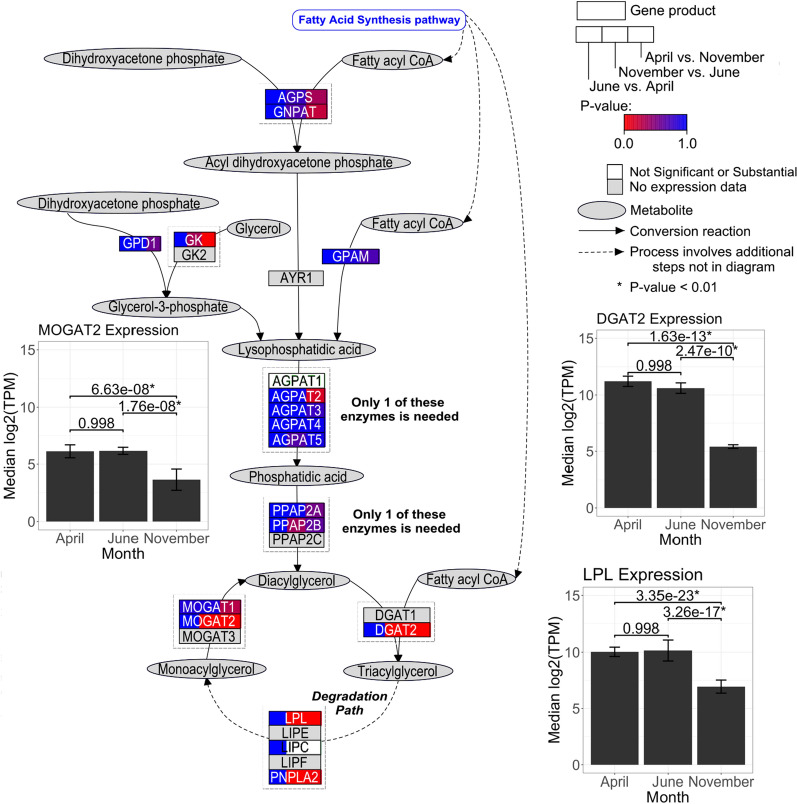
Triacylglycerol synthesis pathway for female seasonal differential expression. Emu triacylglycerol synthesis is represented by the *Gallus gallus* pathway, which is fuelled by the fatty acid synthesis pathway to provide fatty acyl-CoA metabolites. Metabolites (grey oval nodes) are catalysed (black solid arrows) by gene products (rectangle nodes). Dashed arrows represent a process that involves additional steps not displayed in the diagram. Gene products are split into thirds and correspond to the month-wise comparisons from the female seasonal differential expression analysis. Each third is coloured by their respective gene’s Benjamini–Hochberg corrected p-value in a gradient from more significant (red) to less significant (blue). White represents genes that have non-substantial p-values calculated by DESeq2^21^, while grey represents no annotation available for emu or chicken. Three seasonally DE genes (*DGAT2*, *LPL*, and *MOGAT2*) have bar plots of expression levels in median log_2_(TPM) by month featuring median absolute deviation error bars. Numerical Benjamini–Hochberg corrected p-values are shown above the horizontal brackets of the bars that indicate the month-wise comparison, where an asterisk to the right represents statistical significance (p < 0.01).

Of the 100 DE genes, three had a direct metabolic relationship within their respective analyses. To showcase their interconnectivity, a pathway visualization was composed using PathVisio^28^. Considering each differential expression analysis individually, triacylglycerol synthesis exclusively had > 2 DE genes within a pathway in female emus. Chicken triacylglycerol synthesis pathway was used as a model since this species was the closest relative of the emu available in the WikiPathways database^29^ utilized by PathVisio. This pathway features reactions of metabolites catalyzed by enzymes to produce triacylglycerol. Gene products were split into thirds for each monthly comparison and coloured by their Benjamini–Hochberg corrected p-values calculated during female differential expression analysis (Fig. 4). The three female seasonally DE gene expression levels (diacylglycerol o-acyltransferase 2 (DGAT2), lipoprotein lipase (LPL), and monoacylglycerol o-acyltransferase 2 (*MOGAT2*)) for each month were plotted. *DGAT2*, *LPL*, and *MOGAT2* were all expressed significantly lower in November when compared to June (down-regulated) and expressed significantly higher in April when compared to November (up-regulated).

**Figure 5:**
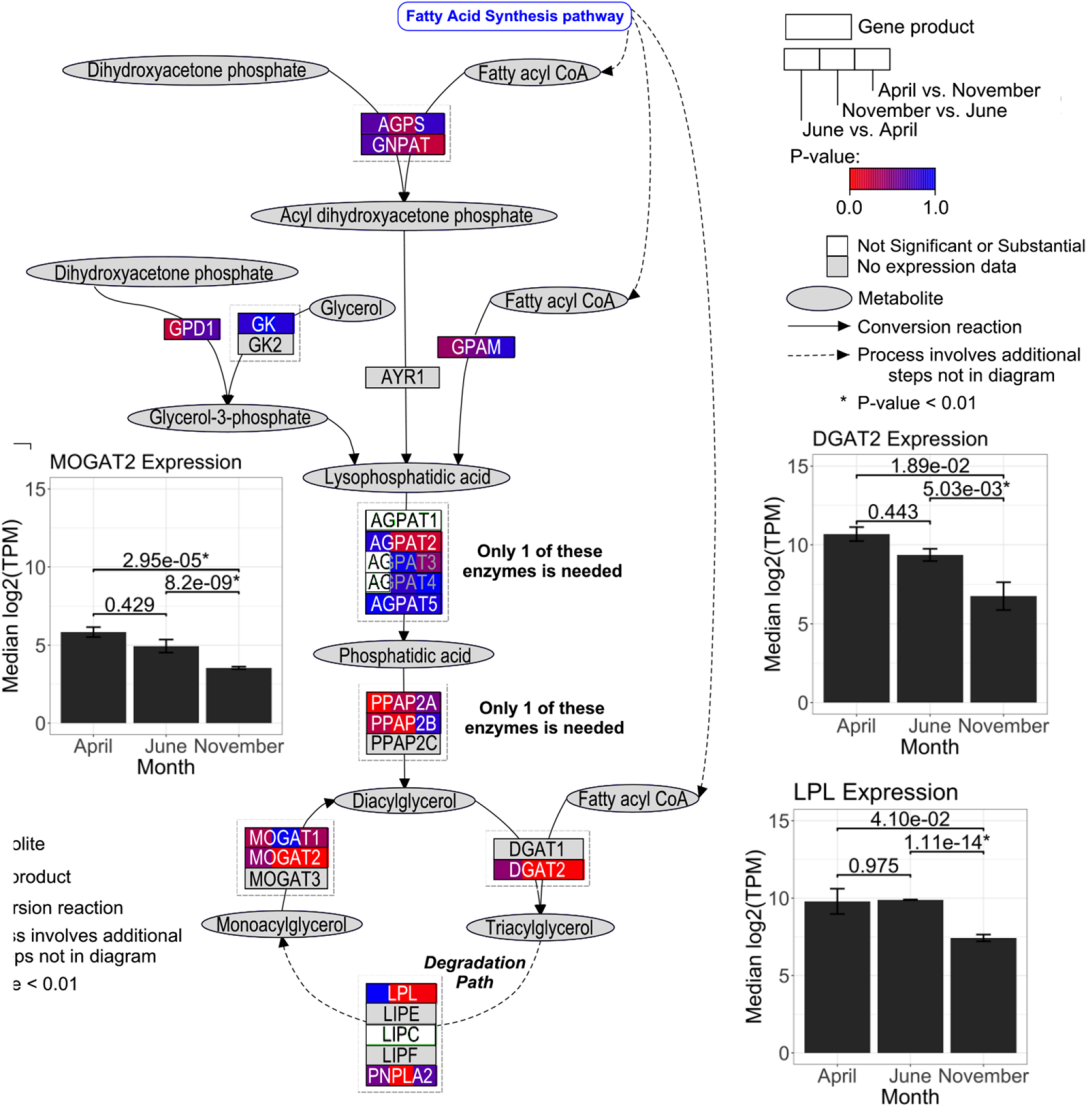
Triacylglycerol synthesis pathway and male seasonal differential expression. Emu triacylglycerol synthesis is represented by the *Gallus gallus* pathway. Gene products are coloured by their respective gene’s Benjamini–Hochberg corrected p-values from the male seasonal differential expression analysis. Three genes (*DGAT2*, *LPL*, and *MOGAT2*) have bar plots of expression levels. *MOGAT2* was seasonally DE in males. Other figure details are the same as in Fig. 4.

To compare the sexes, another triacylglycerol synthesis pathway visualization was composed for male seasonal differential expression (Fig. 5). Expression levels of the same three genes (*DGAT2*, *LPL*, and *MOGAT2*) are displayed, but only *MOGAT2* was significantly DE in males. *MOGAT2* was also expressed significantly lower in November when compared to June (down-regulated) and expressed significantly higher in April when compared to November (up-regulated).

## Discussion

Utilizing RNA-seq has enabled the analysis of emu fat tissue transcriptomes through the changes of seasons and for the comparison of sexes. Nine notable genes were found to affect emu fat metabolism and deposition based on our results, recapitulating related studies. Phenotypically, emu fat pad weights fluctuate annually, so underlying changes in adipose tissue gene expression over time should be apparent^15^. This was supported by our study as we identified 81 seasonally DE genes. When comparing gene ontology profiles of the sexes, only the domain classification of biological processes was significantly different. Gene products can be involved in multiple pathways, indicating they may have essential functions in the tissue^31,32^. Six seasonally DE genes were observed to be involved in multiple enriched pathways, indicating a high involvement in emu fat tissue homeostasis, conceivably causing fat gain and loss. Male and female emus gain and lose fat during the same times of the year, although variability in their fat pad weights have been observed^15^. 19 sexually DE genes were identified potentially explaining the differences observed in fat pad weights.

Immune system process gene ontology category under the biological processes domain was significantly different between the sexes (Fig. 1). Adipose tissue is interconnected to the immune system by communicating the body’s metabolic state to the immune system via adipokines, hormones secreted by the adipose tissue^11^. During starvation, adipose tissue signals the immune system to decrease nutrient consumption by reducing immune cell activation^11^. Male emus go through a period of starvation while incubating eggs, thus, it is plausible that their immune system process is significantly different from females due to their distinct lifestyles during reproduction. The male DE gene, *ITGB2,* generated the significant difference between the sexes in gene ontology profiles. *ITGB2*, also known as *CD18*, is glycoprotein cell adhesion molecule that plays a role in signaling during inflammation that is essential for immune cell recruitment and activation^33^. *ITGB2* is expressed in leukocytes, forming multiple different heterodimers with *CD11* α subunits that function as signal transducer receptors for phagocytosis via complement system, degranulation for release of cytotoxic molecules, and cell adhesion required for leukocyte recruitment^33^. *ITGB2* is a crucial player of the immune defense system. Examination of the effects of miRNAs that bind complementary mRNAs to regulate its gene expression in chicken abdominal adipose tissue preadipocytes and differentiated adipocytes was done in vitro to model adipogenesis^34^. miR-214 was one of the 10 highly expressed miRNAs identified that bound to *ITGB2*, and this pairing had a significant association with abdominal adipocyte differentiation. In a study by Nair et al.^35^, the authors analyzed the gene expression profiles of cultured abdominal subcutaneous preadipocytes/stromal vascular cells to compare the adipose tissue of 14 obese to 14 non-obese non-diabetic Pima Indians. They found *ITGB2* to be one of the 7 inflammation-related genes up-regulated in preadipocytes stromal vascular cells of obese individuals. They hypothesize these up-regulated genes may increase immune cell recruitment to the adipose tissue, resulting in changes of the extracellular matrix for the tissue remodelling needed during adipose tissue expansion in obesity. Considering both our results and supporting studies of *ITGB2* influencing adipose tissue homeostasis, we propose that *ITGB2* alters emu fat in males.

Two genes were identified in females that functioned in multiple enriched pathways: *LPL* and *ANGPTL4*. The adipocyte uptake of dietary fats stored within lipoproteins that circulate the bloodstream requires lipoprotein lipase, the gene product of *LPL*, to hydrolyze the bound triacylglycerol into free fatty acids, resulting in fat storage^9,36,37^. *LPL* has been shown to be strictly regulated for storage and breakdown of fat during the migratory cycle of birds^9^. While birds in migration are selective with their diet, their food choices can affect the regulation of *LPL*^38^. Emus have a seasonal fat gain and loss cycle similar to migratory birds^38^. In mice, *ANGPTL4* promotes the expression of white adipose tissue genes that have functions in triacylglycerol hydrolysis, release of adipocyte intracellular free fatty acids during fasting, and regulation of lipid metabolism^36^. *LPL* activity is regulated by the body’s nutritional state and plays a key role in the mobilization of fatty acids between tissues^37^. In starved rats, it was found that *ANGPTL4* expression increased rapidly while *LPL* activity decreased^37^. In chicken breast muscle, *ANGPTL4* is reported to promote lipid hydrolysis and inhibit *LPL*^39^. These studies suggest that *ANGPTL4* has an inhibitory effect on *LPL*, in concordance with our observations. We observed that *ANGPTL4* was expressed significantly higher in November, when fat is broken down for energy, compared to June, and significantly lower in April, when energy is stored in fat for reproduction, compared to November. *LPL* expression levels were the opposite for those same monthly comparisons. This trend is consistent with *ANGPTL4* promoting lipid breakdown and being expressed inversely to *LPL*. Considering both our results and supporting studies of *LPL* and *ANGPTL4* influencing adipose tissue lipid metabolism, we propose that *LPL* and *ANGPTL4* are involved in the control of the annual cycle of fat gain and loss in Canadian emus.

Four seasonally DE genes were observed to function in multiple enriched pathways for males: *LUM*, *OGN*, *ALDOB*, and *SLC37A2*. Each gene is discussed in the following paragraphs.

It is known that for adipose expansion to occur, increased caloric intake and extracellular matrix remodelling is required^40^. LUM, a small leucine-rich proteoglycan, binds collagen to modulate its fibril formation and plays a role in the structural organization of tissues^40,41^. It was proposed that LUM remodels the extracellular matrix to allow for adipose expansion in order to gain fat^40^. In a study by Yang et al.^14^, RNA-seq was used to investigate DE genes present in chicken thigh muscles affecting the proportion of polyunsaturated fatty acids. The authors found 10 genes associated with fatty acid traits, one of which was *LUM*. Thus, we propose that *LUM* is necessary for remodeling the extracellular matrix during emu adipose expansion.

*OGN*, also known as mimecan, a small leucine‐rich proteoglycan, is secreted extracellularly into the circulation in adipose tissue and induces hypothalamic cytokines IL-1β and IL-6 to inhibit food intake in mice^42,43^. In mice, OGN acts as a satiety hormone to inhibit food intake independent of leptin, which is an adipokine known to control appetite^43^. DE genes in chicken thigh muscles affecting the proportion of polyunsaturated fatty acids were investigated and 10 genes were found that affected fatty acids concentration, one of which was *OGN*^14^. The supporting studies suggest *OGN* may have a function in the regulation of emu fat.

*ALDOB* plays a vital role in glycolysis for energy metabolism. It reversibly converts fructose 1,6-bisphosphate or fructose 1-phosphate into triose phosphate dihydroxyacetone phosphate and either glyceraldehyde 3-phosphate or glyceraldehyde, participating in glycolysis and gluconeogenesis^44^. The sugar glucose is converted via glycolytic pathway into products that can be used for lipogenesis, which is the process of both fatty acid synthesis and fatty acid esterification through which acetyl-CoA is converted into triacylglycerol for storage in fat tissue^44,45^. In a three-way comparison of RNA-seq differential expression analysis, quantitative RT-PCR, and microarray analysis of chicken abdominal fat, *ALDOB* was significantly up-regulated in fatter high-growth chickens that express more lipogenesis genes than low-growth chickens^46^. High-growth chickens up-regulate genes to produce acetyl-CoA to be used in the biosynthesis of cholesterol, fatty-acids, and triacylglycerols^46^. Based on our findings and the related functional research on *ALDOB* suggest that it may aid in emu expression of lipogenesis genes and may have functional relevance in emu fat gain.

*SLC37A2* is glucose-6-phosphate transporter that is expressed highly in macrophages^47^. White adipose tissue is comprised of mainly adipocytes, while the remaining cell population contains macrophages that help maintain white adipose tissue homeostasis, such as by engulfing dead adipocytes for aid in cellular turnover^13,48^. Obesity is a disease of chronic inflammation^13^. It was shown that *SLC37A2* is differentially up-regulated in white adipose tissue macrophages of obese mice compared to wild-type mice^13^. It was also shown that *SLC37A2* negatively regulates adipose tissue macrophage glycolysis and inflammation^13^. It was speculated that higher *SLC37A2* expression in obesity may be a mechanism to mitigate obesity-induced inflammation by decreasing intracellular glucose^13^. *SLC37A2* was shown to be anti-glycolytic, anti-inflammatory, and an anti-oxidative stress regulator as a mechanism to prevent over-production of inflammatory cytokines by managing its glycolytic pathway in mice^47^. Based on our results and the related functional studies, we hypothesize that *SLC37A2* has a role in regulating glycolysis and inflammation in adipose tissue, and may control emu fat deposition and metabolism.

Sexual differential expression analysis revealed November had the most DE genes, possibly due to differences in reproductive lifestyles between sexes. Based on functional studies of the sexually DE genes, two of these genes had functions relevant in fat regulation: *FSHR* and *PLIN2*.

*FSHR* is a G protein coupled receptor for follicle-stimulating hormone (FSH), which regulates reproductive processes^49^. FSH stimulates lipid biosynthesis by upregulating *FSHR* in abdominal adipose tissue of chickens, increasing the accumulation of abdominal fat^49^. In preadipocytes of abdominal adipose tissue treated with FSH, genes functioning in lipid metabolism (*RDH10*, *DCI*, *RARB*, *LPL*, *ACSL3*, and *DGAT2*) were DE when compared to no treatment of FSH^49^. Another study found that polyclonal antibodies that specifically binds FSH to inhibit its action reduced the amount of adipose tissue in wild-type mice and phenocopied the genetic haploinsufficiency for *FSHR*^50^. The aggregate of our results and relevant functional research leads us to propose *FSHR* has functions in stimulating lipid biosynthesis in emu adipose tissue and, hence, influences fat gain and loss.

*PLIN2*, also known as adipose differentiation-related protein, is expressed in adipocytes and is activated when pre-adipocytes differentiate into mature adipocytes^51^. It is a fatty acid binding protein that aids in the uptake of long-chain fatty acids, is a marker of lipid accumulation, and controls adipose tissue homeostasis, thereby affecting body fat distribution^51,52^. In a study in using RT-PCR on *Anas platyrhynchos*, known as Peking duck, *PLIN2* is shown to be expressed highest in abdominal fat^52^. *PLIN2* single-nucleotide polymorphisms were shown to be associated with fatness traits in several chicken breeds^53^. *PLIN2* expression patterns in Sichuan Mountainous Black-bone chicken suggested it is a molecular marker for affecting fat and has an important role in fat development^54^. Additionally, *PLIN2* expression is more abundant in pectoralis muscle of *Dumetella carolinensis*, known as a gray catbird, during spring migration and breeding than late-summer pre-migratory season and autumn migration^55^. Thus, *PLIN2* appears to have a role in influencing emu fat gain and loss.

To functionally relate the DE genes, female triacylglycerol synthesis was visualized (Fig. 4). It is known that LPL hydrolyzes triacylglycerol bound by lipoproteins to monoacylgycerol and two free fatty acids so it can be absorbed into adipose tissue for storage^56,57^. Triacylglycerol is synthesized through two major pathways: the glycerol phosphate pathway and monoacylglycerol pathway^58,59^. In the monoacylglycerol pathway, the seasonally DE gene *MOGAT2* along with other monoacylglycerol acyltransferase isoforms catalyze the conversion of monoacylglycerol with fatty acyl-CoA to diacylglycerol^58^. Diacylglycerol o-acyltransferases including the seasonally DE *DGAT2* catalyzes the conversion of diacylglycerol with fatty acyl-CoA into triacylglycerol^59^. Triacylglycerol is stored in adipose tissue as a long-term energy reserve^12^. The triacylglycerol synthesis visualization highlights the direct relationship of a few DE genes and their interconnected function. In males, in the triacylglycerol synthesis pathway only *MOGAT2* was differentially expressed (Fig. 5). Although *DGAT2* and *LPL* were not differentially expressed in males, they exhibited similar median log_2_(TPM) expression levels to females at each time point.

As a limitation of our study, by using chicken as a reference species rather than the lesser researched emu, we assumed there would be no drastic changes in the molecular functions across these avian species—something that was explored in our previous study^15^, and should be explored further. We confirmed there are underlying changes in adipose tissue gene expression over time that influence the annual fluctuations in emu fat pad weights as evident by the identification of 81 seasonally DE genes. We also demonstrated there are slight differences in gene expression between the sexes, with 19 sexually DE genes identified. These genes may be related to the sexual differences observed in fat pad weight. Our results implicate nine genes (*ITGB2, ANGPTL4, LPL, LUM, OGN, ALDOB, SLC37A2, FSHR, PLIN2)* that may functionally influence fat metabolism and deposition in emus. These nine fat-influencing genes should be further examined in future emu fat research. This study lays the foundation for further downstream studies aimed at improving emu fat production through genomic selection.

## Supplementary Information

Supplementary Information.

## Data Availability

The datasets generated and analyzed during the current study are available in the NCBI Sequence Read Archive repository, https://trace.ncbi.nlm.nih.gov/Traces/sra/sra.cgi?study=SRP336229, SRA ID SRP336229, BioProject ID PRJNA761725.
